# Explosive Vomiting Associated with Proximal Colonic Distention during a Difficult Propofol-Assisted Colonoscopy

**DOI:** 10.1155/2019/6960493

**Published:** 2019-07-17

**Authors:** Kang H. Rah, William Ferges, James Tse

**Affiliations:** ^1^The Rutgers State University of New Jersey, 714 Thistle Hill Lane, Somerset, NJ 08873, USA; ^2^The Digestive Disease Center of New Jersey, 33 Clyde Road, Suite 102, Somerset NJ 08873, USA; ^3^Anesthesiology and Perioperative Medicine, Rutgers Robert Wood Johnson Medical School, 125 Paterson St. CAB 3100, New Brunswick, NJ 08901, USA

## Abstract

We present a case of explosive vomiting associated with the extensive manipulation of the proximal colon during a difficult colonoscopy procedure. The cause of vomiting in this case may have been multifactorial; however, proximal colonic distention was the most likely factor because the onset of vomiting coincided with proximal colonic manipulation and happened without any prodromal signs, coughing, and airway obstruction. Propofol, the sedative most commonly administered to the patient during colonoscopy, allows for a deep state of sedation, and consequently extensive colonic distention and scope manipulation. Colonic distention may lead to a higher risk of vomiting. We reviewed the neurocircuitry associated with vomiting and discussed why proximal colonic distention may increase the risk of vomiting. We emphasize vigilance during the manipulation of the proximal colon because vomiting increases the potential for aspiration pneumonitis and pneumonia in patients under deep propofol sedation with attenuated airway responses.

## 1. Introduction

Propofol sedation performed by an anesthesia provider has been increasingly used for gastrointestinal (GI) endoscopies in the US [1, 2]. Cardiovascular (CV) complications are the most commonly reported complications in such GI endoscopy cases, but they are usually transient and immediately treated [3]. Vomiting and aspiration are relatively underreported but may occur more frequently than currently known [1]. Recent studies have shown that pulmonary aspiration during GI procedural sedation occurs more often than in other procedural sedations [4, 5]. In this case report, we present a patient who vomited explosively during the resection of polyps mostly located in the proximal colon.

The patient discussed below reviewed this case report and gave written permission for the authors to publish this report in a medical journal.

## 2. Case Description

A 62-year-old, 108-kg man with a history of smoking cigars for 30 years, obstructive sleep apnea, benign prostatic hyperplasia, and chronic bronchitis presented for a colonoscopy at the RWJ Endosurgical Center. His previous colonoscopy revealed multiple diverticula involving the entire colon. Additionally, his bronchitis was somewhat exacerbated three days prior to admission. Upon admission, the patient was afebrile but had some residual coughing. Owing to the patient's history of repeated episodes of similar symptoms, we decided to proceed with the planned procedure. The patient was placed on NPO for solid food after midnight, but he took one last dose of SUPREP^R^ (0.2 litres), a bowel cleansing solution, with water (0.3 litres) 6 hours prior to the procedure.

The patient's preinduction vital signs were as follows: blood pressure (BP), 114/72 mmHg; heart rate (HR), 64/min; oxygen saturation (SpO2), 96 %; and respiratory rate (RR), 9/min.

At the start of colonoscopy, the patient received intravenous propofol with supplemental oxygen at 3 L/min via a nasal cannula. Subsequently, the endoscopist used air insufflation for the luminal distention.

Cecal intubation was not easily attainable owing to the extensive diverticulosis and redundancy of the patient's colon. It took approximately 15 minutes to reach the cecum from the splenic plexus, during which time, extensive manipulation of the scope, and application of manual external pressures were required. The endoscopist then proceeded to remove polyps. The endoscopist successfully removed one polyp from the cecum and three from the ascending colon. However, while the fifth polyp was being resected from the transverse colon, the patient suddenly vomited explosively, expelling a slightly yellowish liquid, without any prodromal signs, coughing, and airway obstruction. During this time, the patient's vital signs were stable.

The patient received a total of 200 mg of propofol in incremental doses within 25 minutes prior to vomiting. At the time of vomiting, the patient was quickly put in a head-down position, and his oropharynx was gently suctioned without much vomitus retrieved. His SpO2 decreased from 96% to 92% for about 2 minutes and he experienced some coughing episodes.

Propofol administration was discontinued after the vomiting incident, and the patient was beginning to wake up. His vital signs at this point were stable (BP, 101/55; HR, 66-77; SpO2,91-92; RR 22), and the remainder of the procedure was completed without any incident.

The patient stayed in the postanesthesia care unit for 30 min with stable vital signs and an SpO2 ranging from 93-95% on room-air. He was discharged home with instructions to contact the endoscopist's office or visit the emergency room or urgent care unit in case of any signs of fever, excessive coughing, or shortness of breath. According to the postprocedure follow-up calls, the patient was satisfied with the care given by the team.

## 3. Discussion

The air or carbon dioxide (CO2) insufflation of the colon is used to improve visibility during colonoscopy. However, there is no method for regulating either the volume or pressure of the introduced gas. Patients' discomfort and endoscopists' visibility are the general guidelines affecting the amount of gas insufflated. The maximal pressures generated by endoscopic air pumps range between approximately 300- and 375 mmHg [6].

The patient described in this case report required extensive manipulation of the colonoscope and insufflation of air for a prolonged duration of time for successful cecal intubation owing to the redundancy of his colon and multiple diverticula involving the entire colon. Furthermore, the resection of multiple polyps in the proximal colon (including the cecum, the ascending colon, and the proximal 2/3 of the transverse colon) ultimately led to proximal colonic overdistention. Additionally, the thin wall of the cecum made it more distensible, and high insufflating pressures resulted in more preferential distention of the proximal colon than that of the distal colon. In one study, conventional X-ray revealed colonic dilatations of more than 6 cm in diameter in 71% of room-air insufflation patients, while only 4% of CO2 insufflation patients showed 6 cm dilatation, which suggests that CO2 insufflation may be beneficial in cases involving difficult colonoscopy procedures [7].

The excessive distention of the colonic lumen may induce not only CV complications (hypotension, bradycardia, and arrhythmias), but also vomiting by activating the vagal afferents at the colonic mucosa. The afferent impulses stimulate the dorsal motor nucleus (DMN) through the nucleus tractus solitarius (NTS). The efferent vagus nerve is responsible for the integrated output response to several peripheral organs for emesis [8].

Compared to distal colonic distention, proximal colonic distention may make patients more prone to vomiting owing to the characteristic differences in neurocircuitry between the two parts of the colon. The mechanoreceptors in the proximal colon detect physiological changes (stretching, traction or both), and the afferent vagal fibers send the impulses to the NTS [8–10]. The sympathetic afferent fibers at the proximal colon, as well as the parasympathetic and sympathetic afferents at the distal colon and rectum, sense nociceptive sensations and send impulses to the NTS and other higher central nervous system (CNS) nuclei [8–10]. This neural pathway may explain why vomiting is more prone to occur during proximal colonic distention, since nociceptive impulses may be blunted via propofol sedation, but the physiologic changes could be transmitted through the vagal afferents at the proximal colon.

**Figure 1:**
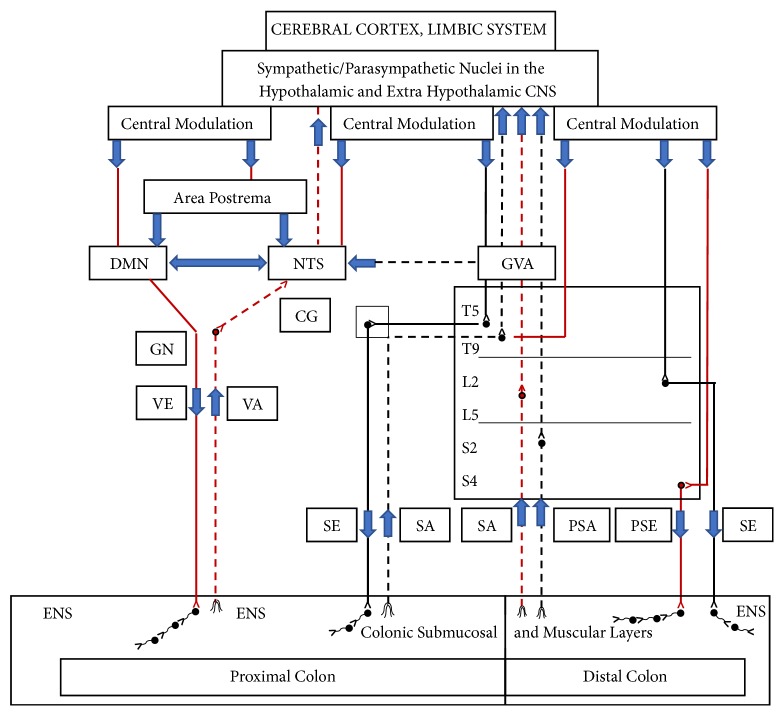
**Sympathetic/parasympathetic neurocircuits of proximal and distal colon**:* DMN* (dorsal motor nucleus),* ENS* (enteric nervous system),* GN* (ganglion nodosa),* GVA* (general visceral afferents),* NTS* (nucleus tractus solitarius),* PSA* (parasympathetic afferent),* PSE* (parasympathetic efferent),* SA* (sympathetic afferent),* SE* (sympathetic efferent),* VA* (vagal afferent), and VE (vagal efferent).* Proximal colon:* vagal efferent fibers (solid red line) travel from the DMN to the submucosal and muscular layers of the proximal colon where they synapse with the multiple neurons and interneurons of the enteric nervous system. Efferent vagal fibers have the two following components: the cholinergic pathway, which has a stimulating effect, and the nonadrenergic noncholinergic pathway, which is inhibitory to colonic motility and secretion. Vagal afferent fibers (dotted red line) originate from the proximal colonic wall and transmit impulses relating to physiological changes (i.e., stretching and distention) to the NTS after synapsing at the GN. The NTS has dense projections that connect to the DMN and area postrema (AP). Impulses from the NTS also projects to higher CNS nuclei for modulation and integration, which provides feedback to the NTS and DMN for stimulation and inhibition. Sympathetic efferent fibers (solid black line) originate from sympathetic nuclei in the thoracic (T5-T9) spinal cord and travel to the proximal colonic wall after synapsing at the celiac ganglion (CG). They induce inhibitory effects except for sphincters. Sympathetic afferent fibers (dotted black line) carry nociceptive information from the proximal colon. After synapsing in the dorsal horn of the spinal cord, they ascend via the spinothalamic tract to reach the NTS directly and via GVAs to reach higher CNS nuclei. Both sympathetic afferent fibers (dotted red line) and parasympathetic afferent fibers (dotted black line) transmit nociceptive impulses from the distal colon via the spinothalamic tract to join GVAs and reach the NTS and higher CNS nuclei. Modulated/integrated information provides feedback to the sympathetic and parasympathetic nuclei in the spinal cord.* Distal Colon:* parasympathetic efferent fibers (solid red line) originate from parasympathetic nuclei (S2-S4) and travel to the distal colon to induce stimulating effects except for sphincters. Sympathetic efferent fibers (solid black line) originate from sympathetic nuclei (L2-L5) and travel to the colonic wall after synapsing at the IMG to induce inhibitory effects except for sphincters.

Impulses from the NTS can directly stimulate the DMN or indirectly stimulate the DMN via modulated and integrated stimuli from higher CNS centers. The DMN transmits efferent impulses to the colon and then to the enteric nervous system in the submucosal area (Meisner's plexus) and muscular layer (Auerbach plexus) resulting in colonic contraction (a homeostatic reflex that relieves distention). This phenomenon is called a vagovagal reflex (Figure 1) [8, 10]. The NTS also sends information to the area postrema (AP) which may stimulate the subnuclei in the DMN responsible for vomiting.

A large-scale study showed that pulmonary aspiration occurs more frequently in colonoscopy patients who undergo anesthesia assistance with propofol [1]. Since not all patients who vomited develop aspiration, the incidence of vomiting during colonoscopy may be much higher than reported. Another study showed that three out of 96 colonoscopy patients under propofol sedation had positive chest scintiscan findings for aspiration. Out of these three patients, one developed pneumonia while the other two remained asymptomatic (1: 3 ratio) [11].

Moreover, propofol sedation attenuates airway responses more than any other sedatives such as midazolam and thus increases the potential for aspiration. This may explain why propofol is associated with higher incidence of aspiration.

Propofol, while being an antiemetic, allows for a deep state of sedation and consequently extensive colonic expansion and scope manipulation. Therefore, colonic distention may lead to a higher risk of vomiting via the aforementioned neural mechanism, particularly when air insufflation is utilized. This speculation has been well-proven in a large-scale study that reviewed over 35,000 anesthesia-assisted colonoscopies, which demonstrated that the incidence of aspiration was more common in cases with anesthesia assistance than in those without anesthesia assistance [1]. Nevertheless, the frequencies of colonic perforation and splenic injury were statistically similar in this large-scale study.

Currently room-air insufflation has remained the standard of care in most endoscopy centers; however, CO2 insufflation may be beneficial in cases involving difficult colonoscopy procedures, particularly in reducing the incidence of vomiting, and the risk of bowel ischemia associated with overdistention of the proximal colon [7].

Proximal colonic distention, however, is not the only cause of vomiting during the colonoscopy. Other possible causes of vomiting associated with colonic distention may include a retrograde spread of gas beyond the cecum into the small intestine in cases of incompetent ileocecal valves, external pressures applied to assist the passage of the scope during a difficult colonoscopy, hypotension, and delayed gastric emptying with various causes. Additionally, passive regurgitation may occur in patients with esophageal motility disorders and incompetent esophageal sphincters when the abdomen is distended. If glucagon is used to relax the colon during a difficult colonoscopy, vomiting may be further accentuated because glucagon-like peptide-1 induces nausea and vomiting owing, at least in part, to actions within the hypothalamus [8].

Additionally, about 95% of the serotonin in the body is found in the GI tract. Colonic distention results in the secretion of serotonin by enterochromaffin cells, which will induce nausea and vomiting [12]. For this reason, we propose the use of 5 HT-3 receptor antagonists for the prevention of vomiting due to colonic distention, especially for patients with risk factors for postoperative nausea and vomiting [13].

When the NTS sends impulses to DMN subnuclei, which are responsible for the CV system, bradycardia and hypotension may develop vasovagal reflex. The reported incidence of the vasovagal reflex varies, but one study reports a 16.5% incidence during colonoscopies [14]. Moreover, in that study, moderate and severe diverticulosis was more commonly associated with the vasovagal group than with the nonvasovagal group.

Anticholinergic medications may be useful in cardiac complication cases (vasovagal reflex) associated with colonic distention, but probably not for the management of vomiting, since maintaining the homeostatic vagovagal reflex may be beneficial.

Therefore, in light of our case results and their possible mechanisms, anesthesia providers should be vigilant and act immediately when prodromal signs of vomiting occur, which may include swallowing, changes in respiratory pattern, licking, retching, and increased activity of abdominal muscles [8]. Anesthesia providers should discontinue propofol administration and advise endoscopists to relieve bowel distention and loosen looped colonoscopes.

When active vomiting occurs, anesthesia providers should apply all basic maneuvers to prevent pulmonary aspiration.

## 4. Conclusion

During colonoscopy under sedation, vomiting occurs more frequently than during other procedures that require sedation [4, 5]. This may lead to serious morbidity and even mortality in patients who are admitted for a relatively simple screening procedure. Vigilance is of the utmost importance, especially during difficult colonoscopies involving the extensive manipulation of the proximal colon.

We discuss the pathophysiology of vomiting resulting from the overdistention or mechanical traction of the proximal colon. In order to reduce overdistention of the colon, the use of CO2 insufflation may be beneficial [6].

The causes of vomiting in our patient may have been multifactorial, but proximal colonic distention combined with the presence of extensive colonic diverticulosis was the most likely contributing factor, since the onset of vomiting coincided with proximal colonic manipulation and happened without any prodromal signs, coughing, and airway obstruction.

Further multicenter studies with large study populations are required to confirm the relationship between proximal colonic distention and vomiting in propofol-assisted colonoscopy so that a method for prevention and treatment is standardized in the field.
